# Parent–Child Relationship Quality and Internet Use in a Developing Country: Adolescents’ Perspectives

**DOI:** 10.3389/fpsyg.2022.847278

**Published:** 2022-02-28

**Authors:** Thao Thi Phuong Nguyen, Tham Thi Nguyen, Ha Ngoc Do, Thao Bich Thi Vu, Khanh Long Vu, Hoang Minh Do, Nga Thu Thi Nguyen, Linh Phuong Doan, Giang Thu Vu, Hoa Thi Do, Son Hoang Nguyen, Carl A. Latkin, Cyrus S. H. Ho, Roger C. M. Ho

**Affiliations:** ^1^Institute for Global Health Innovations, Duy Tan University, Da Nang, Vietnam; ^2^Faculty of Medicine, Duy Tan University, Da Nang, Vietnam; ^3^Youth Research Institute, Ho Chi Minh Communist Youth Union, Hanoi, Vietnam; ^4^Faculty of Social Sciences and Humanities, Hanoi Metropolitan University, Hanoi, Vietnam; ^5^Center of Excellence in Health Services and System Research, Nguyen Tat Thanh University, Ho Chi Minh City, Vietnam; ^6^Institute of Health Economics and Technology (iHEAT), Hanoi, Vietnam; ^7^Bloomberg School of Public Health, Johns Hopkins University, Baltimore, MD, United States; ^8^Department of Psychological Medicine, National University Health System, Singapore, Singapore; ^9^Department of Psychological Medicine, Yong Loo Lin School of Medicine, National University of Singapore, Singapore, Singapore; ^10^Institute for Health Innovation and Technology (iHealthtech), National University of Singapore, Singapore, Singapore

**Keywords:** internet addiction, internet use, parent–child relationship, children, Vietnam

## Abstract

**Objective:**

The goal of the study was to explore the relationship between parent–children relationships related to using the internet among kids and potentially associated factors.

**Materials and Methods:**

A sample of 1.216 Vietnamese students between the ages of 12 and 18 agreed to participate in the cross-sectional online survey. Data collected included socioeconomic characteristics and internet use status of participants, their perceived changes in relationship and communication between parents and children since using the internet, and parental control toward the child’s internet use. An Ordered Logistic Regression was carried out to determine factors associated with parent–children relationship since using the internet.

**Results:**

The characteristics of the relationship between children and their parents since using the Internet were divided into three levels: deterioration (7.0%), stability (78.2%), and improvement (14.8%). The topics that children most often communicate with their parents include learning, housework, and future directions. Two-way interactive activities, such as supporting parents to use the Internet, have a positive impact on the parent–child relationship. Stubborn parental control, such as establishing rules about contact or allowing Internet access and setting up global positioning system (GPS) to track negatively affecting parent–child relationships.

**Conclusion:**

Findings indicated that changes in the quality of the parent–child relationship were self-assessed by participants regard to kids’ internet use, especially in the COVID-19 epidemic context. Educational campaigns and programs to raise awareness of parents as to the dangers and negative influences that their children may encounter online, psychology of children’s behaviors and effects of different responding strategies are recommended.

## Introduction

Using the internet has become one of the most popular leisure-time activities worldwide. In Vietnam, since the internet was officially connected in 1997, internet users sharply increased and reached 68.72 million as of January 2021, equivalent to 70.3% of the population. The average internet use/access time of Vietnamese was 6.5 h every day. Notably, 70.1% of the internet users in Vietnam were reported to be aged 13–34 (Digital 2021: Vietnam: Datareportal, 2021). A 2016 study showed that 72% of Vietnamese aged 15–24 used the internet, of which 74% were reported having the high-risk danger by cyberbullying (The UNICEF, 2017). The popularity of the internet among adolescents poses challenges to parents in protecting their children from excessive internet use and negative influences from the cyber world (Subrahmanyam et al., 2000; Greenfield, 2004). Excessive internet use may exhibit similar patterns and have similar negative consequences to the life of youths and adolescents compared to other addictive behaviors (Shapira et al., 2003; Young, 2004). From the parents’ perspective, the attachment of youths and adolescents to the internet may lead to the passivity in the young people’s daily activities, changes in their psychological states and behaviors, and negative effects on the quality of the parent–child relationship (van Den Eijnden et al., 2010; Shek et al., 2018; Restrepo et al., 2020; Li et al., 2021).

Existing literature argued that parents play a vital role in the management of children’s internet use (Shi et al., 2017). Previous studies found an inverse association between children’s internet problematic use and parental monitoring and bonding (Siomos et al., 2012; Ding et al., 2017). Meanwhile, the relationship between parental control and children’s behaviors appears to be more complicated. Barber et al. (2005) reckoned that parental control could be in the forms of behavioral or psychological control. Behavioral control referred to the use of explicit management strategies by parents including children’s activities supervision, establishing rules, and restrictions to regulate the children’s behavior (Shek, 2005), while psychological control manifested through implicitly adjusting children’s behavior based on their emotions and thoughts. Psychological tactics that were used by parents included guilt infusion, love withdrawal, and authority assertion (Smetana and Daddis, 2002; Rogers et al., 2003). The existing scientific findings have consistently shown that parental psychological control diminished child development and increased parent–child conflicts, while behavioral control positively related children’s developmental outcomes and well-being (Barber et al., 2005; Wang et al., 2007; Bleakley et al., 2016).

Concerning children’s excessive internet use, some studies found that children were less likely to get addicted to the internet if their parents managed firmly in proximity and consistent manner (e.g., establishing the rules for using the internet, finding how to share internet information; Harakeh et al., 2004; Van der Vorst et al., 2005). On the other hand, some empirical evidence showed that the psychological control of parents was positively associated with children’s internet addiction (Giles and Price, 2008; Xiuqin et al., 2010; Cheung et al., 2015). Negative effects of excessive parental control might lead to several mental health issues in children such as low self-esteem (Younes et al., 2016; Shi et al., 2017), depression (Younes et al., 2016; Fayazi and Hasani, 2017), and antisocial behaviors (Barber et al., 2012), which triggered excessive internet use in children as an adapting way for satisfying their psychological needs (Yu et al., 2012). Meanwhile, children who had a high relationship quality with their parents were found to be more likely to have emotional stability, better social competence, and fewer behavioral issues (Schneider et al., 2001). Several publications demonstrated a negative association between adolescents’ internet addiction and having an open parent–child relationship (Zhang et al., 2011; Zhu et al., 2015).

The research landscape concerning internet use behaviors of Vietnamese adolescents has expanded significantly in recent years, in light of the accelerating popularity of the internet among this population (Sobowale et al., 2016; Tran et al., 2017; Zhang et al., 2017; Lan et al., 2020). Furthermore, in the context of COVID-19 pandemic outbreaks, the Vietnamese government implemented proactive measures to prevent spreading, including school closures (La et al., 2020). During the stay-at-home period, spending more time on virtual platforms may influence the parent–children relationship (Cuong et al., 2021). There have been several studies on internet use among youths and adolescents in Vietnam (Sobowale et al., 2016; Tran et al., 2017; Zhang et al., 2017; Lan et al., 2020; Cuong et al., 2021). However, these studies had not offered a comprehensive assessment of the effect of Vietnamese parental management on adolescent Internet use, especially amidst the pandemic. The current study aimed to (1) assess the relationship between parent–children relationships related to using the internet among kids; and (2) identify potentially affected factors.

## Materials and Methods

### Study Design, Sampling Method, and Data Collection

From June to July 2020, an online cross-sectional study was conducted in Vietnam. The eligibility criteria for participating in this survey were: (1) studied from grade 6 to grade 12; (2) currently lived in Vietnam, and (3) agreed to join this study by providing online informed consent. We recruited participants *via* convenience sampling from four cities/provinces undergoing rapid urbanization, including Can Tho, Ba Ria-Vung Tau, Hung Yen, and Thanh Hoa. Participants who suffered from serious illnesses or could not answer questions were excluded from the recruitment process.

The formula to estimate a population proportion was used to calculate the sample size with specified relative precision. With confidence level (%) *α* = 0.05, expected population proportion with better relationship with parent *p* = 34.0% (according to a previous study in Egypt; Moawad and Ebrahem, 2016), and relative precision *ε* = 0.15, the sample size estimated to participate by 332 participants per study sites. The total sample size in four research sites in Vietnam was 1,328. After calculating the total sample size of four research sites, 10% of sample size was added to prevent incomplete survey responses, thus resulting in a total of 1,460 students being recruited. The total of participants who agreed to involve in and completed the questionnaire was 1,216 at the end of data collection period, with the response rate was 83.2%.

### Measurement and Instrument

In this study, we conducted an online questionnaire on SurveyMonkey’s platform^1^. This approach yields efficiencies, such as saving time, reducing cost, and reaching a large of samples. A structured questionnaire including five major components: (1) General characteristics; (2) Parent–child relationship since participants used the internet; (3) Children’s internet use status; (4) Interactive and supportive activities regarding internet use between parents and children; and (5) Social support. A pilot survey was conducted by 10 people for ensuring logicality and adjusting questions with unclear before collecting data. Participants spend 20 min completing the informed consent and questionnaires.

### Variables

#### Outcome Variable

##### Relationship Between Children and Their Parents Since Using the Internet

A question “How the relationship between you and your parents has changed since using the internet?” was used to assess the internet affects family relationships. The response options for this question were divided into three levels: deteriorated, constant, and better relationship.

#### Covariates

##### Socioeconomic Characteristic

We asked participants a number of questions about their information, including gender (male/female), grade (secondary school/high school), current location (urban/rural), current living with (parents/grandparents/siblings), academic performance (excellent/good/fair/moderate/below moderate), and the number of family members that participants can confide in.

##### The Status of Using the Internet Among Participants

Participants were asked to report about time using the internet per day. Additionally, we based on “EU Kids Online 2017” to build four questions about the frequency of using the internet at four places: Family, school, friend’s house, and public areas. For each question, a Likert-seven scale from 1 (Never) to 7 (Almost at the time) was used to collect information from participants (EU Kids Online 2017, 2020). The total score of four questions was summed to assess the frequency of using the internet among students, with the higher score indicated that the frequency of using the internet among participants was higher. The Cronbach’s alpha of these questions was 0.64.

##### Children’s Communication With Their Parents Since Using the Internet

To assess the parent–children communication since using the internet, we asked participants to collect information about children’s attitude with their parents, and the frequency of talking to their parents about daily life. In particular, the question “Do you ignore advice from your parents about using the internet?” (Yes/No) was used to explore the children’s attitude toward their parents. Furthermore, nine popular topics about children’s daily life, such as future orientation, learning, housework, daily living habits, hobby, privacy at home, relationship with friends, the selection of clothing, and the problem of pocket money were developed to assess the parent–child interaction. For each topic, a Likert-five scale from 1 (Never) to 5 (Always) was used to report the frequency of parent communication in daily life. The Cronbach’s alpha of these questions was 0.753.

Based on “EU Kids Online 2017, questions for children and young people” and “Children Online in the European Union 2020” to develop five questions to explore about activities of students with their parents on the Internet (EU Kids Online 2017, 2020; The EU kids online network, 2020), including:

1. Helping parents when they have difficulty using the Internet.
2. Ask for help from parents when the situation is difficult to handle on the Internet.
3. Seek advice from parents on what to do on the Internet.
4. Talk about what you do on the Internet with parents.
5. Talk to parents about what is bothering them on the Internet.
6. For each question, the response options ranged from 1 (Never) to 5 (Always). The Cronbach’s alpha was 0.825.

##### The Parental Management and Care With Internet Use in Children

Based on the survey “EU Kids Online 2017, Questions for children and young people” (EU Kids Online 2017, 2020), a series of questions about parental care and management were developed, including:

###### Parental Management With Internet Use in Children

We developed 10 questions to collect information, including:

1. Rules about how long I am allowed to go online
2. Rules about when I am allowed to go online
3. Knows about what I look at on the internet and the social media
4. Rules about who I can contact on social networks
5. Checks my messages on the social media
6. Checks my information on the Internet
7. Technology to track where I am [such as global positioning system (GPS)]
8. Check out content what I look at on the internet
9. Check out adding my friends and friends list
10. Limits content of what I can see on the internet

###### Parental Care With Internet Use in Children

Eight questions were used to collect data, including:

1. Explains why some online content is good or bad
2. Encourages me to explore and learn things on the Internet
3. Talks to me about what I do on the internet
4. Suggests ways to use the internet safely
5. Helps me when something bothers me on the internet
6. Talks to me about what to do if something online bothers or upsets me
7. Helps me when something is difficult to do or find on the internet
8. Do sharing activities with me on the internet

For all of the above questions, a Likert-five scale was used, with ranging options from 1 (Never) to 5 (Always). Additionally, the Cronbach’s alpha values of the questions about parental management and care were good at 0.857, and 0.859, respectively.

##### Multidimensional Scale of Perceived Social Support

Multidimensional Scale of Perceived Social Support (MSPSS) was a brief, and easy to administer self-report questionnaire which consisted of 12 items. This scale was used to the extent to which an individual perceives social support from three sources: significant others (four items), family (four items), and friends (four items; Wongpakaran et al., 2011). For each item, a seven-point scale from 1 (very strongly disagree) to 7 (very strongly agree) was used (Zimet et al., 2020). The total score of the four items shows that the higher the score, the higher the degree of support. The Cronbach’s alpha was excellent at 0.905.

### Statistical Analysis

Both descriptive and analytical statistics were used to address the main aims of the study by STATA version 16. When screening data, we used the Listwise Deletion method to clean data before analyzing it. Listwise Deletion means that any individual in a data set was excluded from the model if they were missing data on any variable in the analysis. Continuous variables were presented as mean and SD, while categorical variables were presented as frequencies with percentages. In this study, to compare differences in the parent–children relationship among participants, the Kruskal-Wallis test for continuous variables and the *χ*^2^ test were used for categorical variables. Multivariate Logistic Regression was conducted to determine potential predictors of the parent–child relationship among Vietnamese students (Deteriorates = 1; and Constant/Better = 0). To identify potential predictors related to the relationship between parents and children, firstly, we selected according to univariate analysis, and variables with value of *p* ≤ 0.25 were included in the multivariate logistic regression model (Hosmer et al., 2000). Subsequently, these potential covariates could be used in multivariate logistics regression model, including socio-economic characteristics, parent–children communication, parental management and care, and social support. Furthermore, we used Hosmer-Lemeshow test and some criteria, such as R-square, AIC, and BIC to assess the goodness of fit for multivariate logistic regression model and select the best models in this study. These models were then combined with the stepwise forward strategies to reduce models with *p* < 0.2 as the threshold for including variables (Hosmer et al., 2013). A value of *p* < 0.05 was considered statistically significant.

### Ethical Consideration

All procedures performed in studies involving human participants were in accordance with the ethical standards of the Youth Research Institute, Vietnam, and with the 1964 Helsinki Declaration and its later amendments or comparable ethical standards. Online Informed consent was obtained from all participants.

## Results

### Relationship Between Parents and Children Since Using Internet

Table 1 described the characteristics of the relationship between children and their parents since they started using the internet. Participants reported having either deteriorated (7.0%), constant (78.2%), and better (14.8%) relationship with their parents. Participants who reported deteriorating relationships with parents were mainly female (62.4%), high school students (70.6%), living in a nuclear family (92.9%), having an academic performance at fair levels (38.1%). Meanwhile, secondary school students (57.8%), having excellent/good academic performance (68.1%) showed a better relationship with parents. In addition, most people who had a bad relationship with their parents ignored advice about internet use from their parents (91.8%) and had limited internet access (78.8%). People having deteriorated relationship with parents spent significantly more time on the internet compared to those reported having a constant or better relationship. In particular, the internet use frequency of students having deteriorated relationships with their parents was significantly higher than others. The differences were statistically significant with *p* < 0.05.

**Table 1:** Characteristics of relationship between parents and children since using internet.

Characteristics	Parent–children relationships since using the internet						Total		Value of *p*
	Deteriorates		Constant		Better				
	*n*	%	*n*	%	*n*	%	*n*	%	
**Total**	85	7.0	951	78.2	180	14.8	1,216	100.0	
**Provinces**									
Ba Ria—Vung Tau	35	41.2	330	34.7	47	26.1	412	33.9	<0.01
Can Tho	12	14.1	184	19.4	54	30.0	250	20.6	
Hung Yen	20	23.5	206	21.7	25	13.9	251	20.6	
Thanh Hoa	18	21.2	231	24.3	54	30.0	303	24.9	
**Education**									
Secondary school	25	29.4	458	48.3	104	57.8	587	48.4	<0.01
High school	60	70.6	491	51.7	76	42.2	627	51.6	
**Gender**									
Male	32	37.6	446	46.9	95	52.8	573	47.1	0.07
Female	53	62.4	505	53.1	85	47.2	643	52.9	
**Current living**									
Nuclear family	79	92.9	766	80.5	142	78.9	987	81.2	0.05
Extended family	5	5.9	157	16.5	30	16.7	192	15.8	
Others	1	1.2	28	2.9	8	4.4	37	3.0	
**Location**									
Rural	43	50.6	626	65.8	122	67.8	791	65.0	0.01
Urban	42	49.4	325	34.2	58	32.2	425	35.0	
**Academic performance**									
Excellent (≥ 9/10)	11	13.1	143	15.1	59	33.0	213	17.6	<0.01
Good (8–< 9/10)	22	26.2	399	42.1	69	38.5	490	40.5	
Fair (7–< 8/10)	32	38.1	264	27.8	36	20.1	332	27.4	
Moderate and below (<7/10)	19	22.6	142	15.0	15	8.4	176	14.5	
**Number of family members can confide in**									
None	3	3.5	31	3.3	0	0.0	34	2.8	0.19
One	61	71.8	675	71.0	131	72.8	867	71.3	
Two and above	21	24.7	245	25.8	49	27.2	315	25.9	
**Ignore advice of parents**									
No/unknown	7	8.2	350	36.8	101	56.1	458	37.7	<0.01
Yes	78	91.8	601	63.2	79	43.9	758	62.3	
**Parents limit my Internet access**									
No/unknown	18	21.2	333	35.0	63	35.0	414	34.0	<0.01
Yes, a little	37	43.5	498	52.4	84	46.7	619	50.9	
Yes, a lot	30	35.3	120	12.6	33	18.3	183	15.0	
	**Mean**	***SD***	**Mean**	***SD***	**Mean**	***SD***	**Mean**	***SD***	**Value of** ***p***
**Time using the internet per day (hour)**	5.6	3.3	3.9	2.5	4.0	2.6	4.1	2.6	<0.01
**The frequency of using internet** (range: 4–28)	16.5	4.6	14.3	4.4	13.2	4.7	14.3	4.5	<0.01
Family (range: 1–7)	5.5	0.9	5.3	1.1	5.1	1.4	5.3	1.1	0.03
School (range: 1–7)	3.0	1.9	2.3	1.7	2.1	1.4	2.3	1.7	<0.01
Friend’s house (range: 1–7)	3.8	1.8	3.5	1.8	3.0	1.8	3.5	1.8	<0.01
Public area (range: 1–7)	4.2	2.1	3.1	1.7	3.0	1.7	3.1	1.8	<0.01
**Multidimensional Scale of Perceived Social Support (Unit: 1 score)**									
Social support score in friend domain	3.4	1.0	4.0	0.8	4.1	0.9	3.9	0.8	<0.01
Social support score in family domain	3.4	1.0	3.6	0.7	3.8	0.8	3.6	0.8	<0.01
Social support score in other domains	3.7	0.9	3.8	0.8	3.7	0.9	3.7	0.8	<0.01

### Communication and Interaction Between Parents and Children Related to Internet Use

Children’s communication with their parents since using the internet was shown in Table 2. Learning, housework, and future orientation were the topics that students communicated most frequently with their parents. “Helping parents when they have difficulty using the internet” was the most popular activity that children do with their parents on the Internet. The majority of participants having a good relationship with parents showed the highest frequency of communication about all topics, except for pocket money issues. Similarly, with the mutual support in internet use, participants having a good parent–child relationship were higher frequency of interactive and supportive activities than the other groups. These differences were statistically significant with *p* < 0.05.

**Table 2:** Topic, frequency communication and interactive activities regarding internet use between parents and children.

Characteristics	Parent–child relationships since using the internet						Total		Value of *p*
	Deteriorates		Constant		Better				
	Mean	*SD*	Mean	*SD*	Mean	*SD*	Mean	*SD*
**Topics and frequency of parent–child communication (range: 1–5)**									
Learning	2.9	1.1	3.5	0.9	3.9	0.9	3.5	1.0	<0.01
Housework	3.2	1.1	3.3	1.1	3.4	1.3	3.3	1.1	0.14
Future orientation	2.9	1.3	3.1	1.2	3.6	1.3	3.2	1.3	<0.01
Relationship with Friend	2.8	1.0	2.8	1.1	3.3	1.2	2.9	1.1	<0.01
Daily living habits	3.0	1.4	2.9	1.2	3.1	1.3	2.9	1.3	0.04
Hobby	2.3	1.2	2.7	1.2	3.3	1.3	2.8	1.3	<0.01
The selection of clothing	2.4	1.1	2.7	1.1	3.0	1.2	2.7	1.1	<0.01
The problem of pocket money	2.8	1.3	2.4	1.2	2.3	1.2	2.4	1.2	0.01
Privacy at home	2.6	1.4	2.2	1.3	2.7	1.4	2.3	1.3	<0.01
**Interactive and supportive activities regarding internet use between parents and children (range: 1–5)**									
Helping parents when they have difficulty using Internet	3.2	1.3	3.5	1.2	3.9	1.1	3.5	1.2	<0.01
Ask for help from parents when the situation is difficult to handle on the Internet	1.8	0.9	2.1	1.2	2.6	1.4	2.1	1.2	<0.01
Ask for advice from parents on what to do on the Internet	1.5	0.6	2.0	1.1	2.6	1.4	2.1	1.4	<0.01
Talk about what you do on the Internet with parents	1.8	1.0	2.1	1.1	2.7	1.4	2.1	1.1	<0.01
Talk to parents about what’s bothering them on the Internet	1.5	0.8	2.0	1.1	2.5	1.4	2.0	1.1	<0.01

### The Management and Care of Parents With Children’s Internet Use

The parent’s management and care with the children’s internet use were shown in Table 3. People having deteriorated and better relationships with parents showed high levels of parental management with their internet use, particularly with rules about how long (2.9 points)/when they were allowed access internet (2.6 points). For the group having a better parent–child relationship, the highest frequency of parent’s management was controlling what children look at on the internet and social media (2.6 points), and setting the rules of how long/when they were allowed access internet. Less managed activities included checking access contents and friends on the internet, and restricting what they can see on the Internet in all three groups. The differences were statistically significant with *p* < 0.05. Regarding parents’ care with internet use in children, the group having better parent–child relationship gave higher scores about all items than the others, excepted “talks to me about what I do on the internet.” In contrast, the group having deteriorates relationship with parents showed the item of “talks to me about what I do on the internet” reached highest score than the other groups (2.9 points); the remaining items almost had the lowest scores compared to the remaining groups. Overall, people having a better relationship with parents had significantly higher scores on most parental care items questions, except the “Talks to me about what I do on the internet” question. These differences were statistically significant with *p* < 0.05.

**Table 3:** The parent’s management and care with internet use in their children.

Characteristics	Parent-children relationships since using the internet						Total		Value of *p*
	Deteriorates		Constant		Better				
	Mean	*SD*	Mean	*SD*	Mean	*SD*	Mean	*SD*
**Parent’s management with internet use in children (range: 1–5)**									
Rules about how long I am allowed to go online	2.9	1.5	2.5	1.3	2.6	1.3	2.6	1.3	0.14
Rules about when I am allowed to go online	2.6	1.3	2.2	1.2	2.5	1.3	2.3	1.2	<0.01
Knows about what I look at on the internet and social media	2.3	1.1	2.3	1.1	2.6	1.4	2.3	1.2	0.01
Rules about who I can contact on social networks	2.5	1.4	2.0	1.2	2.3	1.4	2.1	1.3	<0.01
Checks my messages on the social media	2.0	1.0	1.6	0.9	1.9	1.2	1.7	1.0	<0.01
Checks my information on the Internet	1.9	1.0	1.7	1.0	2.0	1.3	1.7	1.3	<0.01
Technology to track where I am (such as GPS)	2.4	1.3	1.5	0.9	1.8	1.1	1.6	1.0	<0.01
Check out content what I look at on the internet	1.9	1.1	1.6	0.9	1.8	1.2	1.6	1.0	0.01
Check out adding my friends and friends list	1.3	0.7	1.4	0.8	1.8	1.2	1.5	0.9	<0.01
Limits content of what I can see on the internet	1.6	1.0	1.4	0.8	1.7	1.3	1.4	0.9	<0.01
**Parent’s care with internet use in children (range: 1–5)**									
Explains why some online content is good or bad	3.1	3.2	3.2	1.2	3.4	1.4	3.2	1.4	0.02
Encourages me to explore and learn things on the internet	2.4	1.3	2.8	1.3	3.3	1.4	2.8	1.3	<0.01
Talks to me about what I do on the internet	2.9	1.0	2.6	1.1	2.8	1.3	2.6	1.1	<0.01
Suggests ways to use the internet safely	2.2	1.1	2.5	1.3	2.7	1.4	2.5	1.3	0.03
Helps me when something bothers me on the internet	2.3	1.4	2.4	1.3	3.3	1.4	2.5	1.4	<0.01
Talks to me about what to do if something online bothers or upsets me	2.4	1.2	2.3	1.2	2.8	1.4	2.4	1.3	<0.01
Helps me when something is difficult to do or find on the internet	1.8	0.9	2.2	1.2	2.7	1.6	2.3	1.3	<0.01
Does shared activities with me on the internet	1.9	1.3	2	1.1	2.6	1.2	2.1	1.1	<0.01

### Identifying Factors Related to the Relationship Between Parents and Children Since Using the Internet

Factors related to the relationship between children and their parents since using the internet were shown in Table 4. Individuals in Hung Yen province had a higher likelihood of having deteriorated relationships with their parents (OR = 9.59; 95%CI: 3.25; 28.33), meanwhile, the opposite was true for Thanh Hoa province (OR = 0.18; 95%CI: 0.05; 0.66). Females, high school students, those having ignored advice from using the internet from parents, and those having higher time using the internet per day were more likely to have deteriorated relationships with their parents. In terms of children’s communication with their parents, a higher frequency of talking to parents about learning (OR = 0.29; 95%CI: 0.19; 0.46), selecting of clothing (OR = 0.34; 95%CI: 0.22; 0.51), as well as the higher score of “helping parents when they have difficulty using the internet” (OR = 0.64; 95%CI: 0.45; 0.92), and “ask for advice from parents on what to do on the Internet” (OR = 0.43; 95%CI: 0.23; 0.80) were positively associated with the relationship between children and parents. Regarding parental management and care with internet use in children, higher score of some parent’s activities such as talking to children about what they do on the internet (OR = 1.71; 95%CI: 1.10; 2.66), helps children when something bothers them on the internet (OR = 2.23; 95%CI: 1.46; 3.39), and using technology to track where children are (OR = 6.92; 95%CI: 3.93; 12.16), rules about who children can contact (OR = 1.82; 95%CI: 1.24; 2.68)/when children are allowed to go online (OR = 1.93; 95%CI: 1.28; 2.93), and knows about what I look at on the internet were increased likelihood of having deteriorated relationships between parent and children.

**Table 4:** Logistic Regression for identifying factors related to the relationship between parents and children since using the internet.

Characteristics	The relationship between parents and children since using internet (Deteriorates = 1 vs. Constant/Better = 0)	
	OR	95%CI
**INDIVIDUAL CHARACTERISTICS**		
**Province (Vung Tau—Ref)**		
Can Tho	0.45	0.15; 1.35
Hung Yen	9.59^***^	3.25; 28.33
Thanh Hoa	0.18^***^	0.05; 0.66
**Gender**		
Female	3.78^***^	1.46; 9.77
**Grade (*vs.* secondary school)**		
High school	7.04^***^	2.34; 21.19
**Ignored advice about using the internet from parents (Yes vs. No)**	10.25^***^	2.73; 38.54
**The frequency of using internet (per score)**	1.00	0.90; 1.11
**Time using the internet per day (hour)**	1.32^***^	1.11; 1.57
**PARENT-CHILDREN COMMUNICATION SINCE USING THE INTERNET**		
**Frequency of talking to parents (per score)**		
Learning	0.29^***^	0.19; 0.46
The selection of clothing	0.34^***^	0.22; 0.51
Future orientation	0.78	0.53; 1.14
The problem of pocket money	2.09^***^	1.45; 3.00
**Children’ activities with their parents on the internet (per score)**		
Helping parents when they have difficulty using the Internet	0.64^**^	0.45; 0.92
Ask for advice from parents on what to do on the Internet	0.43^***^	0.23; 0.80
**THE PARENT’S MANAGEMENT AND CARE WITH INTERNET USE IN CHILDREN**		
**Parent’s care with Internet Use in Children (per score)**		
Talks to me about what I do on the internet	1.71^**^	1.10; 2.66
Helps me when something bothers me on the internet	2.23^***^	1.46; 3.39
Helps me when something is difficult to do or find on the internet	0.67^*^	0.44; 1.03
Parent’s management with Internet Use in Children (per score)		
Technology to track where I am (such as GPS)	6.92^***^	3.93; 12.16
Checks my messages on the social media	0.42^***^	0.23; 0.76
Rules about who I can contact on social networks	1.82^***^	1.24; 2.68
Rules about when I am allowed to go online	1.93^***^	1.28; 2.93
Knows about what I look at on the internet and the social media	1.87^**^	1.14; 3.06
Check out adding my friends and friends list	0.13^***^	0.05; 0.29
**SOCIAL SUPPORT**		
Multidimensional Scale of Perceived Social Support		
Social support from family (per score)	0.64^*^	0.38; 1.08

*** *p* < 0.01;

** *p* < 0.05;

* *p* < 0.1.

## Discussion

This research has deepened the understanding of the relationship between parent–child relationship and children’s online behavior. Parental controls or supports of children’s internet use not only affect the behaviors of the children but can also have positive or negative consequences in the relationship between family members. Our results confirmed that parental control behaviors of their children’s internet use might affect the children’s excessive internet use, academic performance results, psychological status as well as the openness of communications and sharing between parents and children. On the other hand, our research found that participants who received encouragement, help, and support from their parents when using the Internet were more likely to establish better relationships with their parents. It also implied that active family communication significantly affected the healthy interaction of Internet access and positive personal behavior.

This study was conducted during the first year of the COVID-19 epidemic in Vietnam; the government took proactive measures to control its spread which included school closures nationwide (La et al., 2020). Children and adolescents were promoted to take classes as well as socialize more online. During the stay-at-home period, especially in the condition of parental supervision, spending more time on virtual platforms can make a young person feel upset, uncomfortable, and the parent–child relationship may become more stressed (Tran et al., 2020; Cuong et al., 2021). Therefore, our results found that a part of participants reported perceiving relationships with parents deteriorated related to using the internet. This issue echoed the previous findings found that children’s internet usage influences the amount of time spent with family (Jackson et al., 2003). Moreover, our study also discovered that young people who had a deteriorated parent–child relationship since using the internet were likely to confide less and ignore parents’ advice. Negative parent–child communication was found to have a significant association with the likelihood of Internet addiction (Xu et al., 2014; Venkatesh et al., 2019). Strict parental rules about internet access may promote compulsive tendencies in children, which leads to the children’s opposition status and decreased frequency of parental communication regarding internet use (van Den Eijnden et al., 2010). Good communication regarding children’s internet use has been found to be a promising tool that parents can use to prevent their teenage children from developing compulsive Internet use (van Den Eijnden et al., 2010). Some studies have also suggested that parent–child communication, which can be enhanced by having appropriate parental care and supports (Svetaz et al., 2014; Jaggers et al., 2015; Shek et al., 2019) may be a protective factor against problematic internet use in adolescents (Kim and Kim, 2003). It is worth noting that, seemingly positive parental control strategies like “talk to children about what they do on the internet” or “help their children when something bothers them on the internet” can be associated with parent–child conflict, as our study found. This may be due to the tendency of young people to express their concerns to people rather than their parents, possibly because of existing lack of parent–child communication (Văn, 2018). Thus, having appropriate parental control strategies is essential in regulating health internet usage in children, avoiding the vicious cycle of negative control—impaired parent–child communication—deteriorated parent–child relationship—unmet psychological needs—more excessive internet use (Wang et al., 2007; Wang and Fredricks, 2014; Lam, 2020).

In Vietnamese society, the notion of “thuong cho roi cho vot,” which means “spanking (with a rod) out of love for the child” suggests that strict discipline was considered a cultural norm for generations (Vu, 2016; Vuong et al., 2018). Therefore, our findings indicated that Vietnamese parents tend to set diehard control strategies such as using GPS tracking on their children’s devices, establishing rules about contacts, or allowing online. The majority of parents regard spending more time on the internet as a distraction from study and a transgression that warrants discipline (Cappa and Dam, 2014). However, the current results proved the diehard parental control strategies negatively influence the parent–child relationship: the higher magnitude and frequency of such control, the worse the parent–child relationship. Negative parental control, which mostly manifests in the form of psychological control through strategies of guilt induction and love withdrawal aiming to make children’ dependent emotion on their parents (Steinberg et al., 1989; Siomos et al., 2012), has been found to harm teenagers’ emotional functioning and the sense of self (Wang et al., 2007), impair the children’ self-esteem and exacerbate developmental difficulties and maladjustment in youths and adolescents (Barber and Harmon, 2002). In contrast, positive parental control in form of behavioral control through monitoring, taking interest in children’s activities, developing disciplinary strategies, and setting regulative rules to regulate children’s behavior has to be associated with positive developmental results in children (Barber et al., 2005; Bleakley et al., 2016).

Changes in the quality of the parent–child relationship were self-assessed by participants showed improvement or deterioration since youngsters used the internet. The mindsponge mechanism, which was proposed by Vuong and Napier (2015) and Vuong (2016), may be used to explain how and why parents “accept or not accept” their kids’ behaviors when using the internet. Parents’ awareness and mindset play crucial factors in accepting children’s behaviors of using online platforms including learning, exchanging, and entertaining *via* the internet. Additionally, belief in their kids is determined as a critical priority card in the mindsponge mechanism because it encouraged parents in promoting support, sharing information, and communicating with their children in participating in social networking activities (Vuong and Napier, 2015). The positive parent–child relationship might provide a solid emotional foundation that encourages positive living and prevents internet addiction in children (Shek, 2010; Floros and Siomos, 2013).

The research findings are of importance for future scholarly works on the parent–children relationship related to using the internet in developing countries, especially in the context of prolonged COVID-19 pandemic. Our findings provide insights for developing evidence-based interventions aiming to lessen the negative impacts of the internet on young Vietnamese people and increase the quality of parent–child communication. First, the intervention programs should prioritize improving parental knowledge, awareness, and attitude about the positive and negative influences of using online platforms (e-learning, exchange, and entertainment), and enhancing parent–child communication. Second, there should be psychological training programs for parents so that they can understand the psychology of children’s behavior and have appropriate responding strategies. Third, a sense of self-responsibility among youths and adolescents should be encouraged through citizen empowerment and emphasizing responsible behavior, so that young people can protect themselves online. Fourth, future studies can benefit from delving deeper into the association between parent–child relationship and children’s internet use by, for instance, exploring the specific roles of father and mother in family relationship quality and their children’s internet use. Last, regardless of the solutions used by parents or educators for reducing the negative effects of the internet on young people and improving the parent–child relationship, it is critical to strictly practice in a disciplined process for an extended period of time until beneficial results are obtained (Vuong et al., 2022).

This study should be viewed in light of its limitations. First, the study employed a cross-sectional design, which did not allow for drawing causal conclusions between parent–children relationship quality and associated factors. Second, the use of an online survey platform might lead to lower response rates as well as lower reliability and validity of the data compared to that of the traditional interview method. Even so, in the context of COVID-19 pandemic and resource-scarce setting of Vietnam, the authors believe that online survey is the optimal approach to reach a large sample size in a short period. Furthermore, in order to limit selection bias, we conducted a pilot survey and tried to present the questionnaire in the most convenient way for participants in the investigation period. Lastly, due to the length constraint on the online platform, we could not measure factors such as individual characteristics of children and parents, or family context; cultural factors inherently influence the internet use (awareness, habits to experiences, and practices). It is recommended that future studies consider evaluating these aspects in order to possibly draw more objective conclusions from the quality of the two-way relationship between parents and children.

## Conclusion

In conclusion, this study found some evidence for a link between the quality of parent–child relationships and internet use behaviors, which might result from general family relations such as quality of parent–child attachment. Participants suggested that their family relationship quality had deteriorated since using the internet, which led to decreasing academic outcomes, loneliness, and depression. Stubborn parental control factors, such as establishing rules about contacts or allowing online, setting up GPS tracking can have a negative impact on the quality of parent–child relationships. Meanwhile, parent-adolescent communication quality, frequency (talking about learning, selection of clothing, and future orientation), and bidirectional interactive activities such as supporting their parents for internet use, affected positively the relationship between children and parents. The present research findings are of importance for future research on parent–children relationship quality and internet use in developing countries. Simultaneously, intervention targets the negative impacts of the internet on young Vietnamese people and increases parent-child communication quality also mentioned in this works.

## Data Availability Statement

The raw data supporting the conclusions of this article will be made available by the authors, without undue reservation.

## Ethics Statement

All procedures performed in studies involving human participants were in accordance with the ethical standards of the Youth Research Institute, Vietnam, and with the 1964 Helsinki Declaration and its later amendments or comparable ethical standards. The patients/participants provided their written informed consent to participate in this study.

## Author Contributions

TTPN, HND, TV, HMD, NN, CL, CH, and RH: conceptualization. TTPN, TTN, LD, and GV: data curation. TTPN and TTN: formal analysis. TTPN, TTN, LD, GV, and SN: methodology. HTD, CL, CH, and RH: supervision. TTPN, SN, LD, and TTN: investigation. TTPN, TTN, GV, and HTD: writing—original draft. TTPN, TTN, HND, TV, KV, HMD, NN, LD, GV, HTD, SN, CL, CH, and RH: writing—review and editing. All authors contributed to the article and approved the submitted version.

## Funding

This study was funded by the NUS Department of Psychological Medicine (R-177-000-100-001/R-177-000-003-001); and NUS iHeathtech Other Operating Expenses (R-722-000-004-731).

## Conflict of Interest

The authors declare that the research was conducted in the absence of any commercial or financial relationships that could be construed as a potential conflict of interest.

## Publisher’s Note

All claims expressed in this article are solely those of the authors and do not necessarily represent those of their affiliated organizations, or those of the publisher, the editors and the reviewers. Any product that may be evaluated in this article, or claim that may be made by its manufacturer, is not guaranteed or endorsed by the publisher.

## Footnotes

^1^ surveymonkey.com
